# Optimization of capsid-incorporated antigens for a novel adenovirus vaccine approach

**DOI:** 10.1186/1743-422X-5-98

**Published:** 2008-08-21

**Authors:** Qiana L Matthews, PingAr Yang, Qi Wu, Natalya Belousova, Angel A Rivera, Mariam A Stoff-Khalili, Reinhard Waehler, Hui-Chen Hsu, Zan Li, Jing Li, John D Mountz, Hongju Wu, David T Curiel

**Affiliations:** 1 Division of Human Gene Therapy, Departments of Medicine, Pathology, Surgery, Obstetrics and Gynecology, and the Gene Therapy Center, University of Alabama at Birmingham, Birmingham, USA; 2 Division of Clinical Immunology and Rheumatology, Department of Medicine, University of Alabama at Birmingham, Birmingham, USA; 3 Department of Experimental Diagnostic Imaging, MD Anderson Cancer Center, University of Texas, Houston, USA; 4 Department of Obstetrics and Gynecology, University of Duesseldorf, Medical Center, Duesseldorf, Germany; 5 Alabama School of Fine Arts, Birmingham, USA; 6 Birmingham VA Medical Center, Birmingham, USA

## Abstract

Despite the many potential advantages of Ad vectors for vaccine application, the full utility of current Ad vaccines may be limited by the host anti-vector immune response. Direct incorporation of antigens into the adenovirus capsid offers a new and exciting approach for vaccination strategies; this strategy exploits the inherent antigenicity of the Ad vector. Critical to exploiting Ad in this new context is the placement of antigenic epitopes within the major Ad capsid protein, hexon. In our current study we illustrate that we have the capability to place a range of antigenic epitopes within Ad5 capsid protein hexon hypervariable regions (HVRs) 2 or 5, thus producing viable Ad virions. Our data define the maximal incorporation size at HVR2 or HVR5 as it relates to identical antigenic epitopes. In addition, this data suggests that Ad5 HVR5 is more permissive to a range of insertions. Most importantly, repeated administration of our hexon-modified viruses resulted in a secondary anti-antigen response, whereas minimal secondary effect was present after administration of Ad5 control. Our study describes antigen placement and optimization within the context of the capsid incorporation approach of Ad vaccine employment, thereby broadening this new methodology.

## Introduction

Adenoviruses (Ad) have recently been employed for a wide range of vaccination strategies [1]. In this regard, a number of practical advantages are recognized in using Ad-based vectors for antigen gene delivery. These advantages include the ease of manipulation of the viral genome, the ability to prepare high titer stocks of recombinant virions, and the ability of the vector to infect a wide array of target cells [2-4] relevant to the achievement of a useful vaccine effect. These considerations highlight the emerging recognition that Ad vectors embody enormous promise for the realization of diverse vaccine interventions. Of note, Ad-based vaccinations have been practically translated for human applications and have progressed in a variety of immunization contexts such as cancer and infectious diseases [5-12].

Currently, new methods to exploit Ad for vaccine purposes have been developed. These recent approaches have utilized the natural mechanisms of Ad virion immunogenicity whereby antigen epitopes can be directly incorporated into the viral capsid as the basis by which immune presentation of the epitope is achieved [10,13-16]. Strategies advancing this "capsid incorporation" paradigm have evaluated a range of virion capsid proteins as well as a variety of antigens, model and pathogenic [10,14-17].

The major capsid protein hexon has been the focus of the majority of these capsid incorporation strategies owing to its natural role in the generation of anti-Ad immune response and its numerical representation vis a via the virion's structural organization [14,18]. Using this strategy, we have developed the means to incorporate heterologous peptide epitopes specifically within the major surface-exposed domains of the Ad capsid protein hexon [18]. Of note, our previous studies have show that we can incorporate small heterologous peptides into Ad hexon hypervariable regions (HVRs) without perturbing viral viability and major biological characteristics such as replication, thermostability, or native infectivity [18]. Other published studies have focused on incorporations at HVR5 or single site incorporations [17]. However, it has been recognized that the ability to place antigen within multiple sites of the hexon capsid protein holds important potential for presenting multiple epitopes/antigens or several copies of the same epitope within a single Ad vector-based vaccine.

In this regard, capsid surface localization of HVR sites derived from X-ray crystallography suggests that both HVR2 and HVR5 loci are potentially useful for capsid incorporation of antigens for vaccination. As noted, there have been recent reports in which HVR5 has been exploited with respect to epitope insertion [10,14,15,18-21]. Based on our abilities to manipulate both HVR2 and HVR5 sites, we sought to explore the relative merits of these two hexon locales. To compare the flexibility and capacities of HVR2 and HVR5, respectively we genetically incorporated identical epitopes of incrementally increasing size within HVR2 or HVR5 of Ad5 hexon. Our study illustrates that hexon incorporated model antigens elicit a varied immune response in the context of antigen placement or antigen size at both the HVR2 or HVR5 locales.

## Materials and methods

### Antibodies

Mouse anti-penta-His_6 _tag monoclonal antibody (34660) was purchased from Qiagen (Valencia, CA). Horse radish peroxidase (HRP)-conjugated goat anti-mouse secondary antibodies were purchased from DakoCytomation (Denmark).

### Cell culture

Human embryonic kidney cells (293) were obtained from and cultured in the medium recommended by the American Type Culture Collection (Manassas, VA). All cell lines were incubated at 37°C and 5% CO_2 _under humidified conditions.

### Recombinant adenovirus construction

In order to generate recombinant adenoviruses with hexon insertions of arginine-glycine-aspartic acid (RGD)-containing sequences, fragments of the Ad5 penton base gene corresponding to the RGD motifs were derived by PCR and cloned into the *BamH*I site in the previously described HVR2-His_6_/pH5S or HVR5-His_6_/pH5S plasmids [18]. The sequences corresponding to penton base-derived peptides, 33RGD, 43RGD, 53RGD, 63RGD, 73RGD, and 83RGD, were PCR amplified from Ad5 genomic DNA with the following pairs of primers: 33RGD sense (s) and 33RGD anti-sense (as), 43RGD sense (s) and 43RGD anti-sense (as), 53RGD sense (s) and 53RGD anti-sense (as), 63RGD sense (s) and 63RGD anti-sense (as), 73RGD sense (s) and 73RGD anti-sense (as), and 83RGD sense (s) and 83RGD anti-sense (as) (Table 1). For additional details, see reference [22]. To create Ad5 vectors containing RGD epitopes in the HVRs of hexon, these resulting plasmids were digested with *EcoR*I and *Pme*I. These resulting fragments containing the homologous recombination regions and the hexon genes were purified, then recombined with a *Swa*I-digested Ad5 backbone vector that lacks the hexon gene, pAd5/ΔH5 [23]. These recombination reactions were performed in *Escherichia coli *BJ5183 (Strategene, La Jolla, CA). The resultant clones were designated Ad5/HVR2-33RGD-His_6_, Ad5/HVR5-33RGD-His_6_, Ad5/HVR5-43RGD-His_6_, and Ad5/HVR5-53RGD-His_6_, all of which contain the green fluorescence protein gene and firefly luciferase gene in the E1 region [18]. The constructs were confirmed by restriction enzyme digestions and sequence analysis. Ad5, Ad5/HVR2-His_6_, and Ad5/HVR5-His_6 _were previously constructed as described [18].

**Table 1:** Primers used in this study.

33RGD -(as)	CGGGATCCTGCTTCGGCCTCAGCGCGC
33RGD -(s)	CGGGATCCGCCGCGGCAATGCAGCC
	
43RGD -(as)	CGGGATCCGGCAGCTTCGGCCGCTG
43RGD -(a)	CGGGATCCAACTCCAACGCGGCAGCC
	
53RGD -(as)	CGGGATCCTTGCGCAGCGGGGGC
53RGD -(a)	CGGGATCCAGCGGCGCGGAAGAGAACTC
	
63RGD -(as)	CGGGATCCCTTCTCGACCTCGGGTTGCG
63RGD -(a)	CGGGATCCAGCAACAGCAGTGGCAGCG
	
73RGD -(as)	CGGGATCCCGGTTTCTTCTGAGGCTTCTCG
73RGD -(a)	CGGGATCCGGTGGCGCAGGCGG
	
83RGD -(as)	CGGGATCCCAGGGGTTTGATCACCGGTTT
83RGD -(a)	CGGGATCCACCGAACAGGGCGGGG
	
3'HVR5-(as)	GGCATGTAAGAAATATGAGTGTCTGGG
5'HVR2-(s)	CTCACGTATTTGGGCAGGCGCC

^a ^antisense, as; sense, s.

^b ^Underlined letters represent the sequences

encoding the RGD motifs.

### Virus rescue and preparation

To rescue viruses, the constructed plasmids were digested with *Pac*I, and 2 μg DNA were transfected (Lipofectamine 2000 Reagent, Invitrogen, Carlsbad, CA) into the Ad-E1-expressing 293 cells. After plaques formed, they were processed for large-scale proliferation in 293 cells. Viruses were purified by double cesium chloride ultracentrifugation and dialyzed against phosphate-buffered saline containing 10% glycerol. Viruses were stored at -80°C until use. Final aliquots of virus were analyzed for physical titer using absorbance at 260 nm. The infectious viral titer (infectious particles per ml) was determined by tissue culture infectious dose (TCID_50_) assay. Modifications of the hexon gene was confirmed by PCR analysis with the primers 5'HVR2 (s), CTCACGTATTTGGGCAGGCGCC and 3'HVR5(as), GGCATGTAAGAAATATGAGTGTCTGGG, which anneal up and downstream of the site of the insertion within the hexon open reading frame (Table 1).

### Whole virus ELISA and sera ELISA

The enzyme-linked immunosorbent assay (ELISA) assay was performed essentially as described previously [24]. In brief, different amounts of viruses ranging from 4 × 10^6 ^to 9 × 10^9 ^VPs were immobilized in wells of a 96-well plate (Nunc Maxisorp, Rochester, NY) by overnight incubation in (per well) 100 μl of 100 mM carbonate buffer (pH 9.5) at 4°C. After washing with 0.05% Tween 20 in Tris-buffered saline and blocking with blocking solution (2% bovine serum albumin and 0.05% Tween 20 in TBS), the immobilized viruses were incubated with anti-penta-His_6 _tag monoclonal antibody (Qiagen, Valencia, CA) for 2 h at room temperature, followed by AP-conjugated goat anti-mouse antibody incubation. Colormetric reaction was performed with *p*-nitrophenyl phosphate (Sigma-Aldrich, St. Louis, MO) as recommended by the manufacturer, and optical density at 450–650 nm (OD_450–650_) was obtained with a microplate reader (Molecular Devices).

For the anti-RGD33-His_6 _and anti-His_6 _response, ELISA plates (Nunc Maxisorp, Rochester, NY) were coated with 20 μM of the RGD33-His_6 _peptide or the His_6 _peptide in 100 μl of 50 mM carbonate (pH 9.6) per well according to the method we described previously [25]. Plates were washed and then blocked with 3% BSA/PBS. After washing, 60 μl of 1:50 diluted sera was added. After incubation for at least 2 hr at RT, the plates were extensively washed, and the isotype-specific HRP-conjugated anti-mouse antibody (Southern Biotech., Birmingham, AL) was added. ELISAs were developed with TMB substrate (Sigma-Aldrich, St. Louis, MO). OD_450–650 _was measured on an Emax microplate reader.

### Mouse immunization

Female C57BL/6J (H-2^b^) mice at 6–8 weeks of age were obtained from the Jackson Laboratory (Bar Harbor, ME). Groups of at least three to five mice were analyzed in each experiment or at each time point. For antibody response analysis, the following adenoviral vectors were injected into each group of mice: Ad5, Ad5/HVR2-His_6_, Ad5/HVR5-His_6_, Ad5/HVR2-33RGD-His_6_, Ad5/HVR5-33RGD-His_6_, Ad5/HVR5-43RGD-His_6_, and Ad5/HVR5-53RGD-His_6 _at 1 × 10^10^viral particles (VPs) per mouse using tail intravenous injection. For CD4^+ ^T cell response analysis, the following adenoviral vectors were injected to each group of mice: Ad5, Ad5/HVR2-33RGD-His_6_, or Ad5/HVR5-33RGD-His_6 _at 1 × 10^10 ^VP per mouse using tail intravenous injection. On day 40, these mice were intravenously boosted with the same dose of the same vectors or peptide. These mice were then sacrificed 9 days later. All animal protocols were approved by the Institutional Animal Care and Use Committee at the University of Alabama at Birmingham.

### Peptide prediction and synthesis

The antigenic epitope of His_6 _and RGD33-His_6 _were predicted using the Emboss program  and by the Kyle-Doolittle hydropathic plot from the FIMM database of functional molecular immunology . Peptide sequences that were given high binding scores in both prediction programs were chosen for ELISA analysis. Peptides were synthesized by GenScript Co (Piscataway, NJ) and were >98% pure as indicated by analytical high-performance liquid chromatography. Peptides were dissolved in 100% DMSO at a concentration of 10 mM and stored at -20°C until use.

### Intracellular flow cytometry staining

Intracellular analysis of cytokines produced by CD4+ T cells was carried out using FACS analysis according to the protocol of Harrington, et al. and Mangan, et al.[26,27]. Briefly, prior to carrying out intracellular cytokine staining, polarized whole spleen cells or CD4+ T cells were stimulated for 5 h with phorbylmyristyl acetate (50 ng/ml; Sigma-Aldrich, St. Louis, MO) and ionomycin (750 ng/ml; Sigma-Aldrich) in the presence of either GolgiStop at the recommended concentrations (BD Pharmingen, San Diego, CA). Cells were first stained extracellularly with fluorescein isothiocyanate-conjugated anti-CD4+ (RM4-5), fixed and permeabilized with Cytofix/Cytoperm solution (BD Pharmingen), and then stained intracellularly with allophycocyanin-conjugated anti-IFN-γ (XMG1.2) and anti-IL-4 (11B11). Samples were acquired on a FACSCalibur (Becton Dickinson, Franklin Lakes, NJ) and data were analyzed with FlowJo (Ashland, OR) software.

### Statistical evaluation

The data are presented as the mean ± the standard error. Statistical analyses were performed with the nonpaired two-tailed Student *t *test, assuming equal variance. Statistical significance was defined as *P *< 0.05.

## Results

### Incorporation of antigenic epitopes within Ad5 hexon HVR2 or HVR5

**Figure 1:**
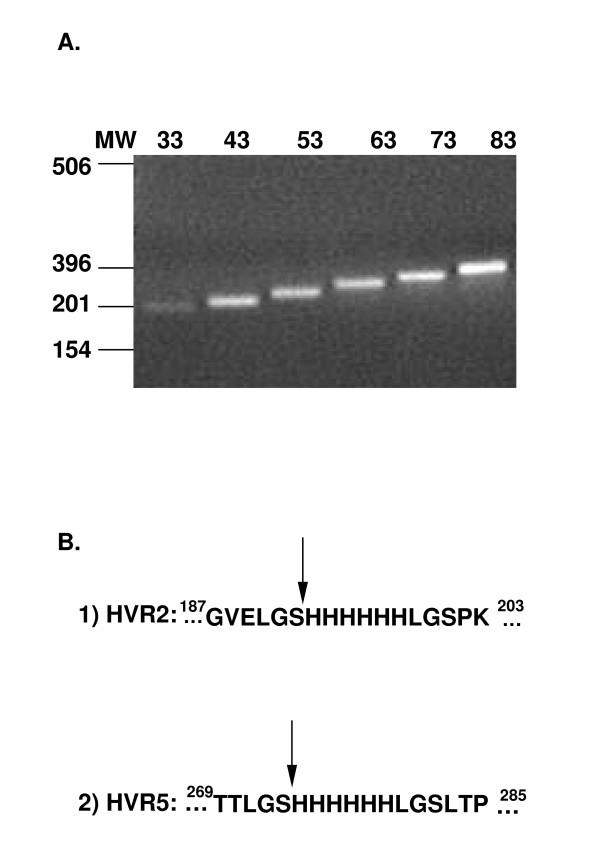
**Construction of hexon modified genomes.** (A) Agarose gel electrophoresis of PCR products were obtained through PCR of genomic DNA of Ad5 using epitope specific primers (Table 1). (B) HVR2 or HVR5 shuttle vector sites which were modified with a RGD motif. The DNA encoding for the respective RGD motifs were cloned into the HVRs of our previously modified shuttle vectors within the LGSHHHHHLGS linker, as indicated by the arrows.

**Figure 2:**
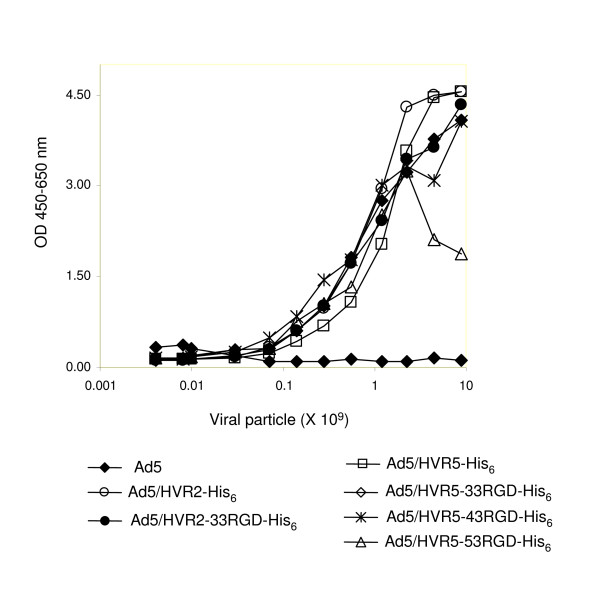
**Model epitopes incorporated in HVRs are accessible in the context of an intact virion.** In the assay, varying amounts of purified viruses were immobilized in the wells of ELISA plates and incubated with anti-His_6 _tag antibody. The binding was detected with an AP-conjugated secondary antibody. These results suggested that the model antigens (tagged with His_6 _epitopes) and the His_6 _epitopes (controls) incorporated into HVR2 or HVR5 were accessible to anti-His_6 _tag antibody at the virion level, indicating that the epitopes were exposed on the virion surface. All of the Ad vectors except Ad5 present His_6_or RGD-His_6_within the hexon. The His_6 _antigenic peptide is presented by Ad5/HVR5-His_6_and Ad5/HVR5-His_6_.

In order to assess the capacity of the Ad5 hexon hypervariable regions to accommodate heterologous polypeptides, we genetically incorporated incrementally increasing fragments of the Arg-Gly-Asp (RGD)-containing loop of the Ad5 penton base. Fragments were engineered to contain the RGD motif in the middle, flanked by penton base-derived sequences of equal lengths on both sides. The length of each flanking sequence in the shortest construct was 15 amino acid (aa) residues; this was increased by 10-aa increments in succeeding constructs [22]. DNA sequences corresponding to the fragments of the penton base protein were assembled by PCR (Figure 1A. and Table 1). These PCR products were cloned between codons for Ser192 and His193 (Fig. 1B–1) of the previously modified Ad5/HVR2-His_6 _genome [18] or between codons for Ser273 and His274 of the previously modified Ad5/HVR5-His_6_genome (Fig. 1B–2) [18]. A total of six fragments encoding the penton base protein ranging in size from 33, 43, 53, 63, 73, and 83 aa were amplified and incorporated into the Ad5 hexon HVR2 or HVR5 region.

### Ad5 hexon HVR2 or HVR5 can accommodate large heterologous polypeptides

The newly designed hexon genes were transferred into the E1-deleted Ad5 genome lacking the hexon gene. Subsequent transfection of 293 cells with the resultant recombinant genomes led to the rescue of 4 of the 12 vectors. Viable viruses were produced with incorporation of 33 aa plus a 12 aa linker at HVR2 or HVR5 (Table 2A). In addition, viable viruses were rescued with incorporations of 43 and 53 aa plus linkers at HVR5 (Table 2A). The recombinant hexon viruses rescued contained the aforementioned penton base-RGD composition (Table 2B). Rescued viruses were further amplified and their identities were confirmed by PCR, non-defective revertant viruses were not detected (data not shown). These data were further confirmed by partial sequencing of the hexon genes contained in the Ad5/HVR2 or Ad5/HVR5 genomes. Having established the identities of the newly rescued Ad viruses, we next tested whether the large incorporations in hexon had any effect on virus stability and/or infectious properties. Physical titer, as well as infectious titer was determined for each virus. The viral particle/infectious particle (VP/IP) ratio was calculated for the control viruses, (Ad5, Ad/HVR2-His_6_, and Ad/HVR5-His_6_) as well as all of the hexon-modified viruses. We observed that, as the incorporation size at hexon increased the VP/IP ratio also increased compared to the His_6 _vectors or unmodified Ad5 (Table 3). A normal VP/IP ratio of unmodified Ad ranges from ~10–30.

**Table 2:** Viable viruses.

**A**.		
**Insert**	**HVR2**	**HVR5**
33RGD Motif + 12 aa Linker	**+**	**+**
43RGD Motif + 12 aa Linker	**-**	**+**
53RGD Motif + 12 aa Linker	**-**	**+**
63RGD Motif + 12 aa Linker	**-**	**-**
73RGD Motif + 12 aa Linker	**-**	**-**
83RGD Motif + 12 aa Linker	**-**	**-**
(+) = viable viruses (-) = not viable viruses.		
		
**B**.		

33RGD motif-	**AAAMQPVEDMNDHAI*RGD*TFATRAEEKRAEAEA**	
43RGD motif-	**NSNAAAAAMQPVEDMNDHAI*RGD*TFATRAEEKRAEAEAAAEAA**	
53RGD motif-	**SGAEENSNAAAAAMQPVEDMNDHAI*RGD*TFATRAEEKRAEAEAAAEAAAPAAQ**	
63RGD motif-	**SNSSGSGAEENSNAAAAAMQPVEDMNDHAI*RGD*TFATRAEEKRAEAEAAAEAAAPAAQPEVEK**	
73RGD motif-	**GGAGGSNSSGSGAEENSNAAAAAMQPVEDMNDHAI*RGD*TFATRAEEKRAEAEAAAEAAAPAAQPEVEKPQKKP**	
83RGD motif-	**TEQGGGGAGGSNSSGSGAEENSNAAAAAMQPVEDMNDHAI*RGD*TFATRAEEKRAEAEAAAEAAAPAAQPEVEKPQKKPVIKPL**	

Core Arg-Gly-Asp (RGD) motif italicized and underlined

**Table 3:** We observed that, as the incorporation size at hexon increased the VP/IP ratio also increased compared to the His6 vectors or unmodified Ad5 .

**Modified Viruses**	**Viral Particle (VP)**	**Infectious Particles (IP)**	**VP/IP**
Ad5	4.58 × 10^12^vp/ml	3 × 10^11^PFU/ml	15.26
Ad/HVR2-His_6_	5 × 10^12^vp/ml	3 × 10^11^PFU/ml	14.7
Ad/HVR5-His_6_	5 × 10^12^vp/ml	4 × 10^11^PFU/ml	14.25
Ad/HVR2-33RGD-His_6_	4.7 × 10^11^vp/ml	2 × 10^9^PFU/ml	236
Ad/HVR5-33RGD-His_6_	1.85 × 10^12^vp/ml	1.58 × 10^9^PFU/ml	1,170
Ad/HVR5-43RGD-His_6_	2.35 × 10^12^vp/ml	3.98 × 10^8^PFU/ml	5,940
Ad/HVR5-53RGD-His_6_	1.01 × 10^12^vp/ml	1.25 × 10^9^PFU/ml	808

### Large epitope incorporations are accessible within Ad5 hexon HVR2 or HVR5

Our previous studies determined that His_6 _epitopes incorporated in HVR2 or HVR5 could bind to anti-His_6 _tag antibody via an ELISA assay, therefore surface exposed [18]. After establishing the ability to place large epitopes into HVR2 or HVR5, we next sought to explore whether the larger epitope incorporations were also surface exposed. Only surface expressed motifs should be accessible to antibody binding, thus, we verified that the RGD-His_6 _motif in HVR2 or HVR5 were accessible on the virion surface by ELISA with an anti-His_6 _antibody (Fig. 2). In the assay, varying amounts of purified viruses were immobilized in the wells of ELISA plates and incubated with anti-His_6 _antibody and appropriate secondary antibody. The results demonstrated that Ad5/HVR2-33RGD-His_6_, Ad5/HVR5-33RGD-His_6_, Ad5/HVR5-43RGD-His_6_, Ad5/HVR5-53RGD-His_6_, and positive controls (Ad5/HVR2-His_6 _and Ad5/HVR5-His_6,_) [18] have significant levels of binding by anti-His_6 _antibody, while negative control Ad5 showed essentially no binding. These results indicate that the RGD-His_6 _epitopes incorporated in HVR2 or HVR5 are exposed on the virion surface.

### Incorporation of epitopes within Ad5 hexon HVR2 or HVR5 elicits an IgG immune response

**Figure 3:**
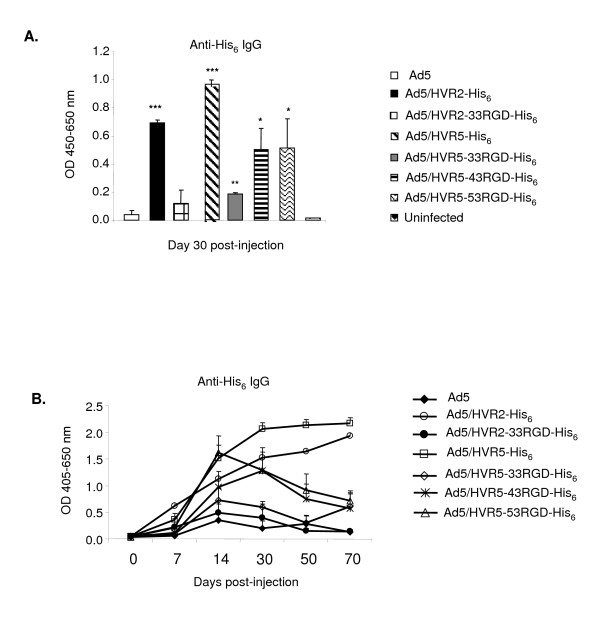
**Capsid-incorporated antigens elicit an IgG immune response.** C57BL/6J mice were immunized with 10^10^VP of Ad vectors. Post-immunization sera were collected after (A) 30 days post-injection or (B) 0–70 days post-injection for ELISA binding assays. 20 μM of synthesized antigenic peptide His_6 _peptide was bound to ELISA plates. Residual unbound peptide was washed from the plates. The plates were then incubated with immunized mice sera, the binding was detected with IgG-specific HRP-conjugated anti-mouse secondary antibody. OD absorbance represents the sera levels of antibodies. Values are expressed as the mean ± standard error of three replicates. * indicates a P value of <.05., ** P < .001, *** P < .00001. Control viruses are Ad5, Ad/HVR2-His_6 _and Ad/HVR5-His_6_.

We next sought to establish that these modified Ad vectors could elicit an immune response in mice. In this regard, a high IgG response is in part indicative of protection for the host organism. Equal amounts of viral particles were used to immunize C57BL/6J mice. The sera from these mice were collected at multiple time points up to 70 days post-injection for analysis with ELISA binding assays (Fig. 3). For these assays synthesized His_6_peptides (His6/linker) (LGSHHHHHHLGS) were first bound to the ELISA plate, the plates were then incubated with immunized mice sera. The binding of mouse anti-His_6 _IgG to the synthesized peptides was detected with a HRP-conjugated secondary antibody (Fig. 3A–B). The data illustrate no binding of the His_6 _peptide with serum from the mice immunized with the negative control Ad5 or the uninfected mice. This is in contrast to substantial binding seen with mice immunized with Ad5/HVR5-43RGD-His_6_, Ad5/HVR5-53RGD-His_6_, or positive controls [18], while Ad5/HVR2-33RGD-His_6 _and Ad5/HVR5-33RGD-His_6 _showed weaker binding. Of note, all of these vectors contain His_6 _epitopes in the modified HVR regions. The data demonstrated that immunization with Ads containing capsid-incorporated antigen elicits an anti-His_6 _IgG response in mice which shows a substantial peak at 30 days (Fig. 3A) when observed over a time course of 70 days (Fig. 3B); furthermore, the magnitude of the immune response was a function of antigen locale and flanking sequences (Fig. 3A–B).

**Figure 4:**
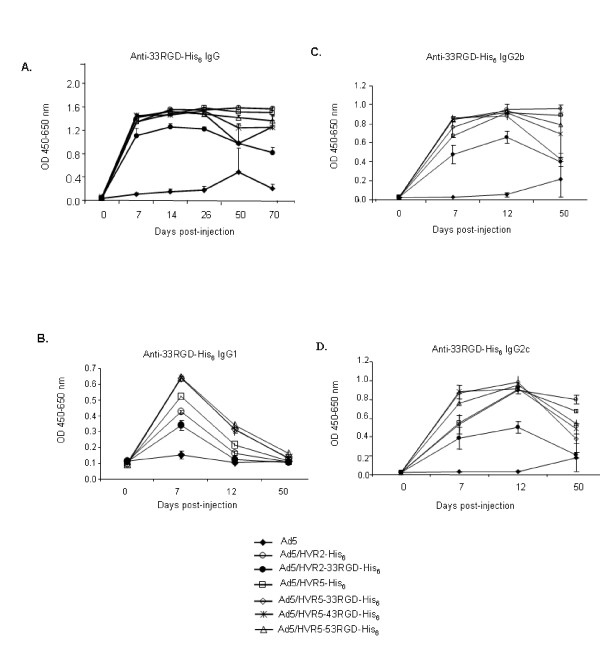
**Capsid-incorporated antigens elicit a varied immune response.** (A-D) C57BL/6J mice were immunized with 10^10^VP of Ad vectors. Post-immunization sera were collected after 50 days post-injection for ELISA binding assays. 20 μM of synthetic peptide 33RGD-His_6_(RGD residue flanked by His_6_/Linker) was bound to the plate. The plates were then incubated with immunized mice sera, the binding was detected with isotype-specific HRP-conjugated anti-mouse secondary antibody (A, IgG; B, IgG1; C, IgG2b; D, IgG2c). OD absorbance represents the sera levels of antibodies. Values are expressed as the mean ± standard error of three replicates. Control viruses are Ad5, Ad/HVR2-His_6 _and Ad/HVR5-His_6_.

Similarly, significant results were seen in response to the synthesized 33RGD-His_6 _antigenic peptide (which contains a core RGD residue flanked by His_6_/linker). These results were observed at time points ranging from 1–70 days (Fig. 4A). Since the probe contained both the 33RGD and His_6 _epitopes binding was expected for all of the sera samples except sera from Ad5 immunized mice.

### Incorporation of epitopes within Ad5 hexon HVR2 or HVR5 elicits a variable humoral immune response

We next performed experiments to determine the quantitative aspects of the isotype-specific humoral responses that were generated in response to our vectors. For IgG1 isotype antibodies, the highest levels of anti-33RGD-His_6 _IgG1 were seen on day 7 after immunization with Ad5/HVR5-33RGD-His_6_, Ad5/HVR5-43RGD-His_6_, and Ad5/HVR5-53RGD-His_6 _virions. These results confirm that the HVR5 loop provides the most immunogenic environment for production of anti-33RGD-His_6 _IgG1 isotype antibodies. Further supporting this, the IgG1 antibody response to the 33RGD-His_6 _in the HVR2 loop was markedly lower when directly compared to the 33RGD-His_6 _in the HVR5 loop (Fig. 4B). The IgG2b (Fig. 4C) and IgG2c (Fig. 4D) isotype specific antibody response to RGD33-His_6 _epitope followed the same pattern as IgG1, except that peak values did not occur until day 12 after immunization, and antibody levels were sustained at high levels out to day 50. These results indicate that RGD-His_6 _epitopes in the HVR5 loop are more immunogenic and invoke higher sera levels of total anti-33RGD-His_6 _IgG antibodies than RGD-His_6 _epitopes in the HVR2 loop.

### Incorporation of epitopes within Ad5 hexon HVR2 or HVR5 elicits a varied T cell and secondary response

**Figure 5:**
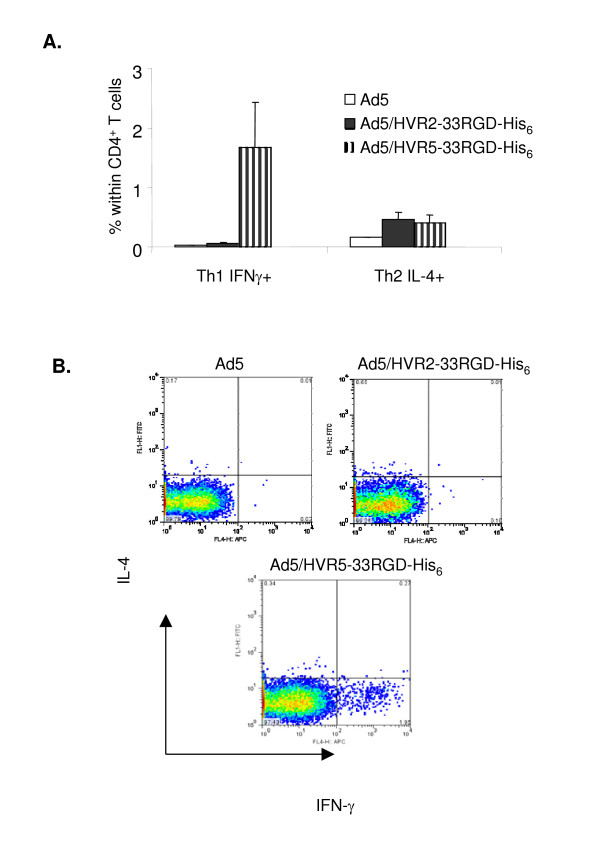
**Capsid-incorporated antigens elicit a varied T cell response.** (A-B) C57BL/6J mice were immunized with 10^10^VP of Ad vectors. On day 40, these mice were intravenously boosted with the same dose of the same vectors. A single-cell suspension of spleen cells was prepared on day 9 after secondary virus infection. Cells were stained with a fluorescent labeled anti-CD4 antibody and then permeabilized in intracellular stain with fluorescent conjugated antibodies against IL-4 or IFN-γ. Samples were acquired on a FACSCalibur and data were analyzed with FlowJo software. Values are expressed as the mean ± standard error of three replicates.

Increased antibody titers of the IgG class require help from either Th1 CD4^+ ^T cells that produce IFN-γ or Th2 CD4^+ ^T cells that produce IL-4 [28]. Th1 is generally associated with isotype class switching to IgG2a (in IgH^d ^strain of mice) or IgG2c (in IgH^b ^stain), whereas Th2 help is associated with class switching to IgG1 or IgG2b in mice [29]. To determine if there is an increase in Th1 or Th2 response to the 33RGD-His_6 _peptide after boost of the Ad5/HVR2-33RGD-His_6 _or Ad5/HVR5-33RGD-His_6 _vector, a single-cell suspension of spleen cells was prepared on day 9 after secondary virus infection. Cells were stained with a fluorescent labeled anti-CD4 antibody and then permeabilized in intracellular stain with fluorescent conjugated antibodies against IL-4 or IFN-γ. CD4^+ ^T cells from mice immunized with Ad5/HVR5-33RGD-His_6 _produced a significant increase in IFN-γ expressing cells and a lesser increase in CD4^+ ^T cells that express IL-4. In C57BL/6J mice immunized with Ad5/HVR2-33RGD-His_6 _or Ad5, there were very low numbers of IFN-γ^+ ^CD4^+ ^T cells (Fig. 5A–B). CD4^+ ^cells expressing IL-4 was equally increased in mice immunized with Ad5/HVR2-33RGD His_6 _or with Ad5/HVR5-33RGD-His_6 _(Fig. 5A–B). The increased IgG antibody response to 33RGD-His_6 _in the HVR5 loop of Ad is associated with a significant increased Th1 T cell response.

**Figure 6:**
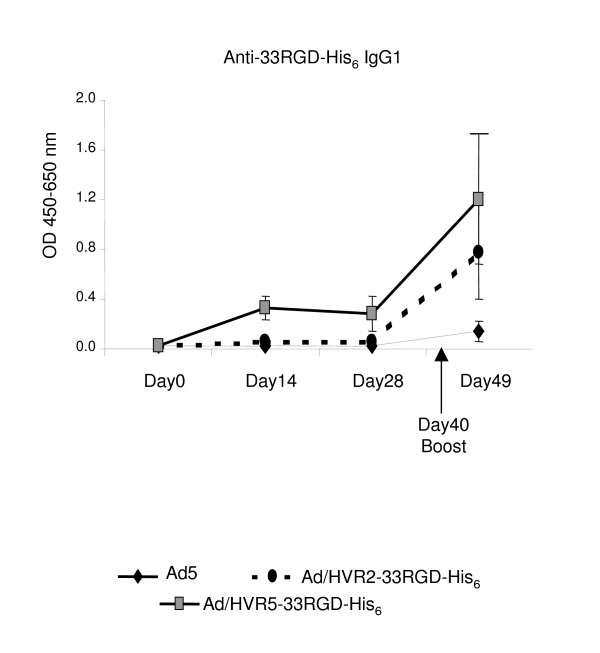
**Repeat administration of hexon-modified viruses results in boosting of the anti-33RGD-His_6 _immune response.** C57BL/6J mice were immunized with 10^10^VP of Ad vectors. On day 40, these mice were intravenously boosted with the same dose of the same vectors. Post-immunization sera were collected after 9 days post-injection for ELISA binding assays. 20 μM of synthetic peptide 33RGD-His_6 _was bound to the plate. The plates were then incubated with immunized mice sera, the binding was detected with isotype-specific HRP-conjugated anti-mouse secondary antibody. OD absorbance represents the sera levels of antibodies. Values are expressed as the mean ± standard error of three replicates.

To evaluate whether immunization with our hexon-modified viruses resulted in improved secondary antibody responses, mice were immunized with Ad5, Ad5/HVR2-33RGD-His_6_, or Ad5/HVR5-33RGD-His_6_. Forty days later, mice were boosted with the respective hexon-modified viruses. Sera levels of antibodies against the 33RGD-His_6 _peptide were determined at day 9 following the booster injection. The Ad5/HVR5-33RGD group exhibited further enhancement with respect to IgG1 antibody response to the 33RGD-His_6 _peptide after boosting compared to Ad5 control (Fig. 6). This trend is similar to that seen after primary immunization (Fig. 4B).

## Discussion

We have developed novel adenovirus vectors that have the potential to optimize adenovirus vaccine approaches. This strategy involves inserting antigenic epitopes of various sizes into HVR2 or HVR5 regions of the Ad capsid protein, hexon, to stimulate epitope-specific antibody responses following vaccination. The ability to insert multiple antigens in the Ad capsid will allow vaccination with antigenic epitopes in one vector. This method offers the ability to compare a range of identical epitopes incorporated within HVRs for antigenic optimization. Our current study is the first study of its kind to compare a range of identical epitopes incorporated within HVRs for antigenic optimization. Importantly, our data ascribe a maximal antigenic incorporation size at HVR2 and HVR5 as it relates to identical antigenic epitopes.

Similar studies have been performed by other groups, Worgall and colleagues describe incorporations of a neutralizing epitope from the *Pseudomonas aeruginosa *outer membrane protein F (OprF) into adenovirus HVR5 [10]. The authors showed an increase in antibody response in BALB/c mice consisting of both IgG1 and IgG2a subtypes. Additionally, when mice immunized with the virus containing the OprF epitope were subjected to pulmonary challenge with *P. aeruginosa*, 60 to 80% survival was achieved. This was in contrast to results seen by McConnell et.al, who published that chimeric hexons containing incorporations of B. *anthracis *protective antigen (PA) elicited antibodies against PA in mice but failed to yield protection against anthrax toxin (lethal factor) challenge [15]. The authors speculate that the varying results reflect a difference in the ability of the selected epitopes to elicit a neutralizing response in the varying disease models or a difference in the antibody titers necessary to achieve protection against *P. aeruginosa *compared to lethal factor challenge. In addition, they speculate that the latter may be related to the fact that in the anthrax model the response is directed against a secreted bacterial toxin, while in the *P. aeruginosa *model the response is directed against the bacterium itself. Similar studies have been performed by Krause et. al, [14]. Krause's study compared the immune response generated by incorporating the hemagglutinin (HA) protein of the influenza A virus incorporated into the outer Ad capsid protein hexon, penton base, fiber knob, or protein IX. The HA epitope was recognized by the anti-HA antibody in all four modified virions with slightly stronger binding to the HA presented in hexon HVR5. However, this study does not investigate whether the size of the incorporated epitopes could also affect the immune response generated.

The strategy we pursued involved the genetic incorporation into hexon HVR2 and 5, respectively. We chose the RGD-containing motif to incorporate into the hexon protein because the RGD motif has been demonstrated to have a critical role in Ad entry. Thus by incorporating this molecule into the Ad hexon we speculated that it might be possible to enhance Ad viral tropism. [17]. In addition, we have previously established that these RGD motifs can be inserted into another Ad capsid protein fiber, thus modulating vector tropism [22]. A total of six fragments of the penton base protein ranging in size from 33 to 83 aa were incorporated into the Ad5 hexon HVR2 or HVR5. Viable viruses were produced with incorporations of 33 aa at HVR2 and up to 53 aa at HVR5 (Table 2A). To effectively invoke an epitope-specific immune response, genetically incorporated epitopes must be accessible on the Ad surface. This study illustrates that RGD-His_6 _motifs incorporated within HVR2 or HVR5 were accessible on the adenovirus surface based on anti-His_6 _ELISA (Fig. 2). There was no significant difference between *in vitro *antibody binding of viruses that contain His_6 _residues at HVR2 or HVR5, or viruses that contain the 33RGD-His_6 _epitope at HVR2 or HVR5. This finding confirms that the 33RGD-His_6 _motifs incorporated within HVR2 or HVR5 are indeed accessible on the Ad surface and should therefore be available to antibodies *in vivo*. We observed that increasing the size of incorporations at hexon HVRs increased the virological viral particle/infectious particle ratios (Table 3), we speculate that virus assembly and stability is affected. In addition, we have observed more aggregation with inserts incorporated at the Ad hexon HVR5 locale, we also further speculate that insertions containing RGD epitopes lend to virus aggregation. Since modifications to Ad capsid proteins can influence infectivity as well as immunogenicity of Ad vaccines and transduction efficiency, it is possible that our modifications would significantly alter the infectivity of Ad. Ad infectivity occurs through the binding of the Ad capsid proteins penton base and fiber to cellular receptors [30-33]. More recently, hexon HVR's have been implicated in liver transduction [19-21]. We speculate that these recent findings by kalyuzhniy and colleagues, indicate that our Ad vectors are more clinically relevant due to the likelihood of less liver transduction.

Successful stimulation of immune responses by Ad vaccines schemas are thought to be dependent partly on the activation of antigen presenting cells, particularly dendritic cells [34,35]. Indeed, genetic modifications made to the capsid in this present study impair some virological properties such as virus particle/infectious particle ratios and gene transfer efficacies (data not shown), but our data indicates that *in vivo *immune response was not affected. However, we will pursue investigation regarding the uptake of our hexon-modified virus by antigen presenting cells. Of note, in this study we notice higher *in vivo *immune response of viruses containing 43 or 53 RGD-His_6 _epitopes at HVR5 compared to that of 33RGD-His_6. _Sequence analysis of these three epitopes show no obvious reason for this trend (ie. hydrophobic or hydrophilic patterns), therefore; detailed structural analysis must be performed.

Finally, our results indicate that mice boosted with Ad5/HVR2-33RGD-His_6 _or Ad5/HVR5-33RGD-His_6 _produced an improved secondary immune response as compared to the control Ad5 vector (Fig. 6). Successful boosting is an important factor because anti-Ad exposure after administration of Ad vectors does not generally allow repeat administration with an Ad vector of the same serotype [36-40,10]. Anti-Ad immunity is thought to be an obstacle for the use of Ad as a gene therapy vector; re-administration of the same vector would be beneficial in the development of Ad-based vaccines to enable boosting of antigen-specific immune response. In our study, repeat immunization resulted in boosting of the anti-33RGD-His_6 _antibody responses. The Ad5/HVR5-33RGD-His_6 _vector exhibited the highest antibody response to both 33RGD-His_6 _peptide and His_6 _peptide (data not shown) after boosting; therefore the Ad5/HVR5-33RGD-His_6 _vector is the best construct to generate the Ad vaccine response with respect to our model antigens.

Our study in contrast, to other reports illustrates the qualitative differences with respect to incorporation of large epitopes within HVR2 or HVR5, until now most reports only investigate HVR5 as a potential incorporation locale. Our study demonstrates that HVR5 is more permissible than HVR2 with respect to incorporation of our largest model antigen. Immunizations with vectors that present smaller His_6 _insertions at HVR2 compared to HVR5, yield similar results with respect to antibody response and insertion locale. In contrast, immunizations with viruses containing large insertions at HVR5 yielded higher antibody and Th1 responses compared to insertions at HVR2. These results were in contrast to that seen with *in vitro *ELISA assays, which were equal binding of insertions at HVR2 or 5 independent of insertion size (Fig. 2). Furthermore, it is likely that large insertions at HVR2 are not permissible due to the surrounding Ad protein structure/environment. However, smaller inserts may be tolerated at HVR2.

We plan to investigate factors limiting insertions at HVR2 and HVR5 by means of cryoEM analysis, this work will correlate well with the Ad crystal structure and cryoEM analysis which has been recently solved [41-44]. In the aggregate, our study demonstrates that utilization of the HVR2 or 5 locales predicate optimal antigen size and configuration. Based on this technology, we will be able to establish the critical correlates between antigen locale/accessibility within the capsid context and vaccine efficacy. Our study evaluated model antigens at HVR2 or HVR5; further studies are necessary to evaluate therapeutic antigens at these locales in the context of binding and antibody neutralization. Transitioning our dual hexon presentation platform to present therapeutic antigens will also allow us to evaluate and use challenge models for efficacy and antigen protection assays. Capsid incorporation of antigens is a highly innovative strategy to present antigens in the context of adenovirus vaccine schemas. This strategy can also be exploited to construct multivalent vaccines, which can allow vaccination against multiple strains of a particular infectious disease or protection against multiple unrelated diseases. Of particular interest to us is the potential to expand our dual hexon antigen presentation strategy to develop Ad-based vaccinations against HIV infection and many other infections or diseases.

## Abbreviations

Ad: Adenovirus; Ad5: Adenovirus serotype 5; aa: Amino acid; ELISA: Enzyme-linked immunosorbent assay; HA: Hemagglutinin; His: Histidine; His_6_: Six-histidine; HVRs: Hypervariable regions; IP: Infectious particles; RGD: Arg-Gly-Asp; VP: Viral particles.
